# Weaknesses out of strength: PNA against the penicillin-binding proteins

**DOI:** 10.1016/j.omtn.2025.102828

**Published:** 2026-01-19

**Authors:** Mateusz Wdowiak

**Affiliations:** 1 University of Warsaw, Centre of New Technologies, Stefana Banacha 2c, 02-097 Warsaw, Poland

## Main text

According to the World Health Organization (WHO), diarrheal illnesses affect approximately 550 million people annually, including 220 million children under the age of 5 years. The non-typhoidal *Salmonella* is among the four major global causes of such illnesses and, due to its adaptability to frequently used drugs, is considered a high-priority drug-resistant pathogen.^1^ Therefore, there is a pressing need to develop novel antibacterial strategies targeting *Salmonella* infections. A recent study, published in *Molecular Therapy Nucleic Acids* by El-Fateh et al., describes the elimination of *Salmonella* spp. by targeting the genes encoding its major penicillin-binding proteins (PBPs).^2^ Using newly designed peptide-nucleic acid (PNA) sequences conjugated to the cell-penetrating peptide (KFF)_3_K as a carrier, the authors show that these antisense conjugates effectively eliminate bacteria both *in vitro* and in an animal infection model. Introducing (KFF)_3_K-PNA conjugates into a *Salmonella* spp. infection treatment in *Caenorhabditis elegans* may be an important step toward subsequent testing in mammalian systems and, ultimately, the clinical application of PNA therapeutics.

*Salmonella* spp. is a group of Gram-negative, foodborne bacteria belonging to the *Enterobacteriaceae* family. As one of the most frequent causes of gastrointestinal infections, the abundance of anthropogenic factors favors the exposure to high doses of antibiotics and the rapid development of resistance mechanisms.^3^ Among the most important are PBPs - peptidoglycan synthesis and crosslinking enzymes. PBPs are targeted by β-lactam antibiotics; their inactivation results in the inhibition of bacterial cell-wall synthesis and cell death. However, mutational alterations in PBPs conferred resistance development by reducing antibiotic binding at the active site or by acquiring β-lactamase-like activity.^4^ As a result, the rapid increase in extensively drug-resistant *Salmonella* strains underscores the urgent need for novel, specific antimicrobial agents.

Peptide nucleic acids (PNAs) are DNA analogues that retain the canonical nucleobases. Compared to the canonical nucleic acids, the deoxyribose-phosphate backbone is replaced by a pseudopeptide backbone composed of modified glycine units linked by amide bonds.^5^ This structural modification determines enhanced resistance to both proteolytic and nucleolytic degradation. Moreover, substitution of the negatively charged deoxyribose-phosphate backbone with a charge-neutral glycine backbone increases the binding affinity of PNAs for complementary nucleic acids by reducing electrostatic repulsion between the molecules. These properties render PNAs as promising agents for antisense therapy against bacterial infections, where bacterial viability is suppressed by inhibiting essential gene expression. However, effective antisense activity requires direct interaction of the antisense oligonucleotide (ASO) with its target mRNA (or DNA) in the cytoplasm. Cellular uptake of PNAs constitutes a major limitation due to two principal factors: (1) their large molecular size, typically exceeding ∼600 Da, which precludes passive diffusion through porins, and (2) unfavorable hydrophobic and electrostatic properties.^6^ Although the neutral charge of the PNA backbone is advantageous for binding kinetics, it concurrently imposes a limitation on intracellular transport, as unmodified PNAs are unable to traverse bacterial membranes without assistance. The first carrier described to enable efficient PNA uptake was the cell-penetrating peptide (KFF)_3_K. Cationic, membrane-active peptides are confirmed as effective carriers for PNAs, primarily owing to their electrostatic interactions with the negatively charged lipopolysaccharides of the bacterial outer membrane.^7^ Despite this breakthrough in PNA delivery, the majority of further studies included PNA sequences targeting the same genes, emphasizing the acyl carrier protein (*acpP*) gene.^8^

El-Fateh et al. employed this validated approach to turn *Salmonella* Typhimurium drug-resistance factors into an antisense-therapy target. Their study included three new PNA sequences, targeting the start codons in PBP-coding genes *ftsI*, *mrcB*, and *mrdA*, and four scrambled sequences. Each of them was conjugated with the (KFF)_3_K peptide to improve the water solubility and permit the transport to the cytoplasm. The antimicrobial efficacy of the conjugates was then tested using complementary approaches, including the bacterial killing protocol (performed according to the minimal inhibitory concentration assay standards) and the quantification of mRNA expression using an RT-qPCR approach. Antimicrobial testing confirmed a high efficacy of (KFF)_3_K-PNA conjugates in *Salmonella* elimination, with the reduction of bacterial titer by > 3log (>99.9%) (Figure 1 from El-Fateh et al.). It estimated the MIC values of <2 μM (Figure 2 from El-Fateh et al.). The quantitative RT-qPCR indicated the reduction of the target genes relative expression by ∼50% (Figure 3 from El-Fateh et al.). Among the tested conjugates, the anti-*mrdA* provided the best results.

In the next stage of the study, the authors investigated morphological changes in bacterial cells, following exposure to the peptide-PNA conjugates, using inverted microscopy imaging (Figure 4A from El-Fateh et al.). Cells treated with the anti-*ftsI* conjugate exhibited a considerable elongation and filamentation, consistent with defective cell division. The anti-*mrcB* conjugate also interfered with cell division; however, disruption appeared to occur at a later stage, likely during daughter cell separation. The treatment with the most effective conjugate, anti-*mrdA*, resulted in the formation of spherical cells, indicative of severe perturbations at early stages of cell wall synthesis. Such profound structural defects may account for the significantly enhanced antimicrobial activity observed for this conjugate. At the same time, none of the conjugates caused significant membrane damage, as assessed by membrane integrity assays (Figure 4B from El-Fateh et al.).

A particularly notable aspect of the study by El-Fateh et al. was the translation of the *in vitro* validated compounds to an *in vivo* model of *Salmonella* spp. gastrointestinal infection in *C. elegans*. This approach enabled simultaneous assessment of the safety of orally administered PNA conjugates in an animal model and their antimicrobial efficacy *in situ*, where attainment of an effective intracellular concentration represents a major challenge. *In vivo* treatment resulted in an almost complete (∼100%) reduction in bacterial titers in infected worms at the tested concentrations, yielding efficacy comparable to that of the reference antibiotic gentamicin (Figure 5 from El-Fateh et al.). Antimicrobial evaluation was complemented by a 7-day fitness and survival assay. Untreated *Salmonella* infection led to nearly complete mortality of the worms, whereas treatment with each of the three PNA-peptide conjugates—anti-*ftsI*, anti-*mrcB*, and anti-*mrdA*—significantly improved host fitness and effectively resolved the infection. The anti-*mrdA* conjugate exhibited the strongest protective effect, with survival rates comparable to those observed for gentamicin at equivalent concentrations (∼50%). In contrast, none of the scrambled-sequence conjugates conferred a measurable survival benefit within the 7-day post-infection period (Figure 6 from El-Fateh et al.).

Looking forward, this study provides strong encouragement to move beyond the stage of *in vitro* investigations and advance the development of novel antimicrobial agents toward whole-animal model testing, with the longer-term goal of clinical application. Notably, targeting drug-resistance determinants with sequence-specific antisense conjugates may yield two complementary therapeutic benefits: direct bacterial eradication or resensitization of resistant strains to conventional antibiotics. Consequently, such conjugates could be employed either as standalone antimicrobials or in combination with existing antibiotics or antimicrobial peptides. The exclusive use of a single carrier type - (KFF)_3_K conjugated to multiple PNA sequences - by El-Fateh et al. highlights opportunities for further optimization, including the evaluation of alternative transporters and other cell-penetrating peptides as delivery vehicles. In addition, future studies could assess the efficacy of these conjugates against clinical, multidrug-resistant *Salmonella* isolates, as well as other clinically relevant members of the *Enterobacteriaceae* family.

## Declaration of interests

The author declares no competing interests.

## Declaration of generative AI and AI-assisted technologies in the writing process

During the preparation of this work, the author used ChatGPT version 5 in order to improve grammar and language. After using this tool/service, the author reviewed and edited the content as needed and takes full responsibility for the content of the publication.
